# Targeting mitochondrial dynamics by AZD5363 in triple-negative breast cancer MDA-MB-231 cell–derived spheres

**DOI:** 10.1007/s00210-023-02477-7

**Published:** 2023-04-24

**Authors:** Yingqiang Fu, Wei Dong, Yuting Xu, Lin Li, Xin Yu, Yuheng Pang, Liujia Chan, Yuhan Deng, Cheng Qian

**Affiliations:** 1 Department of Breast Cancer Surgery, Harbin Medical University Cancer Hospital, Harbin Medical University, Haping RD NO, 150086 Harbin, Heilongjiang Province People’s Republic of China; 2 North China Translational Medicine Research Center of Harbin Medical University, Harbin Medical University, Harbin, 150086 Heilongjiang China

**Keywords:** 3D stem cell spheres, Mitochondrial fusion, AKT inhibitor, Drug resistance

## Abstract

Breast cancer stem cells (BCSCs) have been suggested to contribute to chemotherapeutic resistance and disease relapse in breast cancer. Thus, BCSCs represent a promising target in developing novel breast cancer treatment strategies. Mitochondrial dynamics in BCSCs were recently highlighted as an available approach for targeting BCSCs. In this study, a three-dimensional (3D) cultured breast cancer stem cell spheres model was constructed. Mitochondrial dynamics and functions were analyzed by flow cytometry and confocal microscopy. We have demonstrated that the protein levels of FIS 1 and Mitofusin 1 were significantly increased in BCSCs. Moreover, Capivasertib (AZD5363) administration could suppress Mitofusin1 expression in BCSCs. Our use of MitoTracker Orange and annexin V double-staining assay suggested that AZD5363 could induce apoptosis in BCSCs. The sensitivity of stem cell spheres to doxorubicin was investigated by CCK8 assay, and our results indicated that AZD5363 could re-sensitize BCSCs to Doxo. Flow cytometry analysis identified doxo-induced CD44 and CD133 expression in BCSCs could be suppressed by AZD5363. In combination with AZD536, doxo-induced apoptosis in the BCSCs was significantly increased. In conclusion, our study explored, for the first time, that AZD5363 could target mitochondrial dynamics in 3D cultured stem cell spheres (BCSCs) by regulating Mitofusin.

## Introduction

Regardless of recent advances in therapeutic developments, breast cancer remains to be one of the most common types of cancer and a major cause of mortality worldwide (Schilsky et al., 2020). Breast cancer stem cells (BCSCs) are a small subset of cancer cells that drive tumor initiation, progression, drug resistance, and metastasis (Yang et al., 2017; Butti et al., 2019). Therefore, BCSCs are a promising target for developing effective strategies that against breast cancer (Butti et al., 2019). Recent studies have indicated that enhanced mitochondrial metabolism is a novel feature and therapeutic target of cancer stem cells (CSCs) (Chen and Chan, 2017; Sessions and Kashatus, 2021; Naik et al., 2022).

Mitochondria coordinate several physiological cell function and are highly dynamic and undergoing constant fusion and fission (Pernas and Scorrano, 2016). Increasing amounts of evidences has indicated that alterations in mitochondrial dynamics in cancer cells contribute to various aspects of tumorigenesis and cell progression (Vyas et al., 2016; Burke, 2017). Additionally, emerging research has indicated that cancer stem cells rely heavily on mitochondrial dynamics for chemotherapeutic resistance and stemness maintenance (Zhou et al., 2017; Kumar et al., 2021). Therefore, targeting the mitochondrial dynamics could be a potential therapeutic approach for cancer stem cell treatment.

The AKT signaling pathway was suggested to play a pivotal role in the regulation of breast cancer stemness and metastasis (Bozorgi et al., 2015). In previous studies, AZD5363, a known AKT inhibitor, was reported to suppress anti-cancer agent induced stemness in breast cancer cell lines (Zhu et al., 2021). Here, we investigated the potential effects of AZD5363 on three-dimensional (3D) cultured breast cancer stem cell spheres. Our study suggests that mitochondrial dynamics and function could be enhanced in 3D cultured breast cancer stem cell spheres, and that the AKT inhibitor AZD5363 has the potential to suppress stemness, and re-sensitizing BCSCs to chemo drugs by regulating mitochondrial fusion. Our findings also support future pursuit of AZD5363 as a therapeutic agent target for BCSCs.

## Materials and methods

### Reagents and chemical

Doxorubicin (Doxo, purity>98%) was purchased from Sigma-Aldrich (St. Louis, MO, USA). AZD5363 (Capivasertib, purity>98%) was purchased from Selleckchem (Shanghai, China). An annexin V-FITC staining Apoptosis Detection Kit (BD Biosciences Pharmingen, San Diego, CA, USA) was used to detect apoptotic cell death. MitoTraccker Orange CMTMRos (Thermo Fisher Scientific, USA) was used to evaluate mitochondrial activity. Primary antibodies used as follows: anti-FIS antibody, anti-Mitofusin-1 antibody, PE-conjugated anti-CD133 antibody, FITC-conjugated anti-CD44 antibody, and APCC-conjugated anti-CD24 antibody, were all purchased from Biolegend (San Diego, CA, USA). Fluorescent conjugated secondary antibodies, APC Donkey anti-mouse IgG, and PE Donkey anti-rabbit IgG, which were used in confocal and flow cytometry analysis were purchased from Biolegend (San Diego, CA, USA). Hoechst 33342 was purchased from Thermo Fisher Scientific (Waltham, MA, USA). DAPI was purchased from BD Pharmingen (Franklin Lakes, NJ, USA).

### Cell culture and 3D stem cell spheres formation

Human triple-negative breast cancer cell line MDA-MB-231 was purchased from ATCC, and cultured in DEME/F12K medium with 10% fetal bovine serum and in aired with 5% CO2 at 37 °C. The 3D stem cell culture was based on a semisolid Matrigel^TM^ system according to a previous publication (Bahmad et al., 2018). In brief, single cell suspensions that obtained from cultured MDA-MB-231 cells were resuspended in a 1:1 mixture of Matrigel (BD Biosciences), DMEM/F12K medium containing B27 (Thermo Fisher Scientific, Inc., Waltham, MA, USA), epidermal growth factor (EGF, 20 ng/ml; Solarbio Science and Technology Co., Ltd., Beijing, China), and basic fibroblast growth factor (bFGF20 ng/ml, Solarbio Science and Technology Co., Ltd., Beijing, China), before being plated in 6-well plates at a density of 5000 cells/well. Cells were then cultured with medium as described for 14 days, and the medium was changed every 3 days. After that, the cultured spheres were retrieved using Matrigel Recovery Solution (BD Biosciences) as per the manufacturer’s protocol. The stemness was detected by flow cytometry after staining with CD24, CD44, and CD133. The 2D cultured MDA-MB-231 cells as described were used for control.

### Cell viability assay

Cell viability was evaluated by using CCK8 assay. In brief, cells (obtained from 2D cultured MDA-MB-231 cell or 3D cultured breast cancer stem cell spheres (BCSCs)) were seeded in 96-cell plates (4000 cells/well) and cultured overnight before being treated with different concentration of doxorubicin (0, 25, 50, 100, 200, 400, 600 nM), as well as the same concentrations of Doxo combined with 5 μM AZD5363, for 48h. After adding 10 μL of CCK8 solution, the plates were incubated at 37°C for 2–4h. The plates were then measured at 450 nm using a full-wavelength microplate reader (#Mutiskan GO, USA).

### Immunofluorescence for confocal observation and flow cytometry detection

Single-cell suspensions that obtained from 2D cultured MDA-MB-231 cells or BCSCs were seeded in a confocal dish (for confocal observation) or low-attachment 6-well plates (Corning, Oneonta, NY, USA) at a density of 10,000 cells/well for flow cytometry detection. Cells were cultured overnight before being treated with Doxo (50 nM), AZD5363 (5 μM), and Doxo plus AZD536 for 48 h. After that, the cells were then fixed for 15 min at room temperature with paraformaldehyde (2%) buffer, and permeabilized with Triton X-100 (0.05% in PBS) for 15 min. After triple washing with cell staining buffer (BD, NJ, USA), cells were incubated with anti-FIS antibody (1:50 for flow cytometry detection, and 1:100 for confocal observation) and anti-Mitofusin-1 antibody (1:50 for flow cytometry detection, and 1:100 for confocal observation) primary antibodies for 30 min followed by incubation with anti-mouse APC(1:50), or anti-rabbit PE(1:50) for 30 min before being detected by confocal microscopy, flow cytometry (FACS Calibur, BD), and an ImageStreamX MkII instrument (Amnis, Luminnex), as well as being analyzed with IDEAS Software accordingly.

### MitoTracker orange staining assay

Single-cell suspensions that obtained from 2D cultured MDA-MB-231 cells or BCSCs were seeded in 6-well plates and cultured overnight before being exposed to AZD5363 with the final concentration of 0, 1, 2, and 5 μM, or Doxo (50 nM), or Doxo plus AZD536, for 48 h. After incubation, the cells were collected, washed triple times with ice-cold PBS and resuspended in 400μL of MitoTracker orange staining solution (Thermo Fisher Scientific, CA, USA) in the dark at 37 °C for 20 min. After the described staining, a total of 10,000 cells per sample was counted, and analyzed with an FACS Calibur flow cytometer. Mitotracker Orange CMTMRos and Hochest 33342 double-stained cells were also observed by using image flow cytometry (ImageStreamX MkII instrument, Amni, Luminex).

### Apoptosis assays

An Annexin V-FITC apoptosis kit from BD Pharmingen (Franklin Lakes, NJ)) and Hoechst 33342 were used to detect apoptosis cells according to the manufacturer’s instructions. Single-cell suspensions that obtained from BCSCs were seeded in low-attachment 6-well plates at 1 × 10^5^ cells per well. Cells were then exposed to AZD5363 with final concentrations of 0, 1, 2, and 5 μM, or Doxo (50 nM), AZD5363 (5 μM), or Doxo plus AZD536, for 48 h. After incubation, cells were collected, washed three times with ice-cold PBS, and resuspended in 100 μL of binding buffer. Then, 5-μL annexin V-FITC and 5-μL Hoechst 33342 were added and incubated in the dark at 37 °C for 20 min. After the described double staining, a total of 10,000 cells per sample was counted, and analyzed with a FACS Calibur flow cytometer and image flow cytometry (ImageStreamX MkII instrument, Amni, Luminex).

### Colony formation assay

For the colony formation assay, single cells obtained from BCSCs were seeded at 400 cells per well in a 6-well plate and cultured for 24 h before being treated with Doxo (20 nM), AZD5363 (50 nM), and Doxo plus AZD5363 for 48 h. Then, they were exchanged with fresh medium and cultured for 14 days. The clones were stained with 1% crystal violet solution (Solarbio, Beijing, China) for 15 min. After washing with PBS for several times until the crystal solution was washed away, the colonies were imaged by camera

### Statistical analysis

The bioassay results were expressed as means ± standard deviation (SD). At least three samples were prepared for assays of every attribute. Data analysis was performed using an ANOVA test and unpaired *t* test. A *P* value less than 0.05 was considered as statistically significant.

## Results

### The breast cancer stem cell spheres formation from MDA-MB-231

**Fig. 1:**
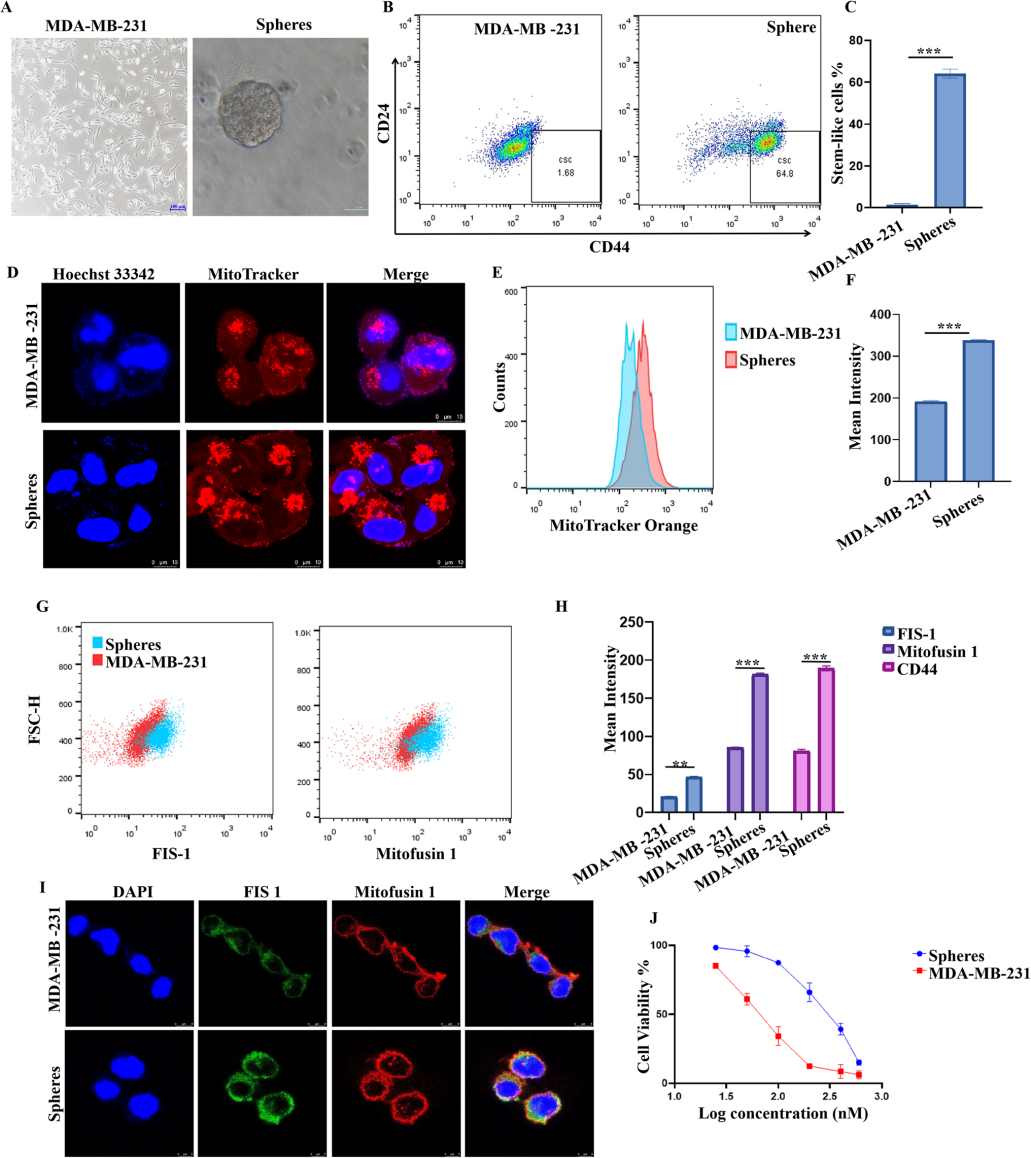
Mitochondrial dynamics was enhanced in 3D cultured spheres derived from MDA-MB-231 cells. **A** Morphological characterization of MDA-MB-231 cells and 3D cultured stem cell spheres by light microscopy. **B**, **C** Date from flow cytometry analysis revealed that CD24^low^CD44^high^ stem cell were enriched in 3D cultured stem cell spheres. **D** Images from confocal microscopy observation displayed that mitochondria activity was enhanced in stem cell spheres compared to MDA-MB-231 cells. **E**, **F** MitoTracker orange was used to identify mitochondria activity in MDA-MB-231 cells and 3D cultured stem cell spheres, data from flow cytometry was displayed as overlapped histogram map (E) and mean intensity was calculated (F). **G**, **H** Mitochondrial fission and fusion markers, FIS 1 and Mitofusin 1, in MDA-MB-231 cells and 3D cultured stem cell spheres were also detected. The results were displayed in overlapped dot plot (G), and mean intensity was calculated (H). **I** Expression of FIS 1 and Mitofusin 1 in MDA-MB-231 cells and 3D cultured stem cell spheres was also observed by confocal microscopy. **J** Cell viability of different concentration of doxorubicin-treated MDA-MB-231 cells and 3D cultured stem cell spheres was compared. The stem cell spheres were more resistant to doxorubicin. * *p* < 0.05, ** *p* < 0.01, *** *p* < 0.001, *n*=3

We have generated breast cancer spheres based on a 3D semisolid Matrigel^TM^ system from triple-negative breast cancer MDA-MB-231 cells, as displayed in Fig. 1A. To analyze the stemness of the cultured spheres, flow cytometry was performed after staining with FITC-CD44, and APC-CD24. Figure 1B shows that the percentage of the CD24^low^CD44^high^ stem cell population in 3D cultured spheres was enriched from 1.4%±0.46 to 64.1%±2.2 (Fig. 1C).

### Mitochondrial activity was increased in stem cell spheres

Mitochondrial activity and distribution patterns were evaluated using MitoTracker orange staining assay. As we can see from confocal images as showed in Fig. 1D, MitoTracker orange intensity was considerably higher in stem cells than in MDA-MB-231 cells. The fluorescence intensity was also detected by flow cytometry (Fig. 1E, F), with the result agreeing with the confocal observation. The mean intensity increased from 191.7±1.5 in MDA-MB-231 cells to 337.7±1.7 in the stem cell sphere group.

### Mitochondrial dynamics were enhanced in stem cell spheres

Mitochondrial dynamics are regulated by two opposed processes: mitochondrial fusion and fission. In this study, we evaluated the mitochondrial fission protein FIS-1, and mitochondrial fusion protein Mitofusin 1, as well as CD44 expressions in BCSCs by using flow cytometry detection and confocal observation. As we showed in Fig. 1G, and H, FIS-1, Mitofusin 1, and CD44 were all markedly increased in spheres, while the mean intensity increased from 20.6±0.4, and 81±1.9 to 46.7±0.6, and 181.7±3.2, respectively. The increased expression of FIS-1 and Mitofusin-1 was also observed by confocal microscopy, as showed in Fig. 1I. We further examined the cytotoxicity of doxorubicin towards MDA-MB-231 cells and 3D culture stem cell spheres. As the cell viability curve that we displayed in Fig. 1J, stem cell spheres were more resistant to doxorubicin.

### AZD5363 suppressed Mitofusin expression in stem cell spheres

**Fig. 2:**
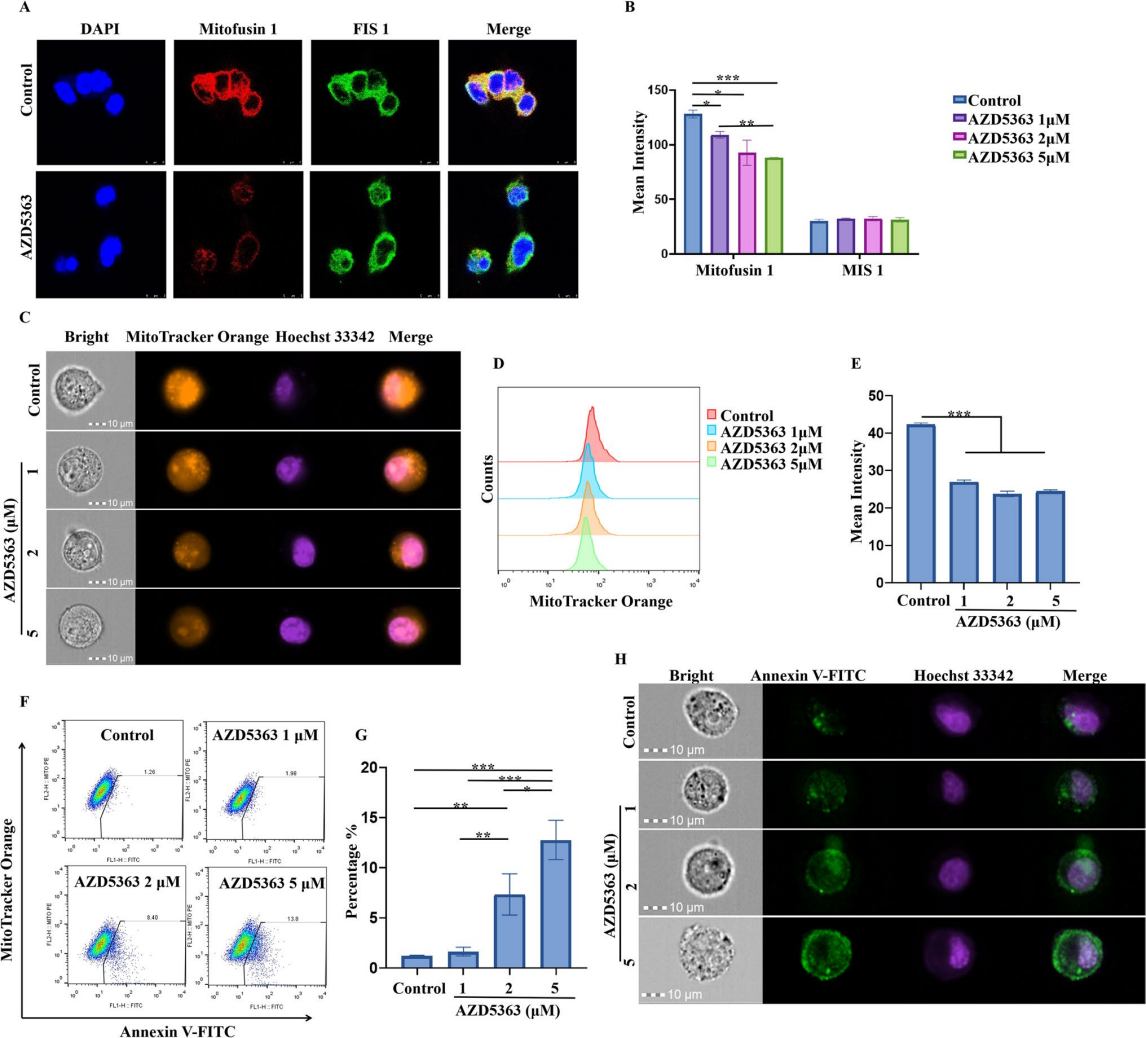
AZD5363 could target mitochondrial dynamics in 3D cultured stem cell spheres by regulating of mitofusin. **A** Expression of FIS 1 and Mitofusin 1 in 3D cultured stem cell spheres treated before and after AZD5363 was observed by confocal microscopy. **B** Flow cytometry detection indicated that Mitofusin 1 expression could be inhibit by AZD5363 in concentration-dependent manner in stem cell spheres. **C** A imaging flow cytometry was used to detect MitoTracker orange in stem cell spheres treated with different concentration of AZD5363. **D**, **E** Half overlapped histogram plot and column plot revealed that fluorescence intensity of MitoTracker orange in stem cell spheres could be decreased by AZD5363. **F**, **G** Annexin V-FITC and MitoTracker double staining assay revealed that proportion of apoptotic cells with lower mitochondral activity could be increased by AZD5363 in a concentration dependent manner. **H** AnnexinV-FITC and Hoechst 33342 double staining assay conformed that apoptosis could be induce by AZD5363 in stem cell spheres. * *p* < 0.05, ** *p* < 0.01, *** *p* < 0.001, *n*=3

Previous publications indicated that AKT activation contributed to cancer stem cell (CSC) traits, such as self-renewal, migration, and chemo-resistance. Additionally, the AKT pathway was proposed to play a key role in mitochondrial fusion and fission dynamics. Thus, in our study, we further investigated the potential effects of AKT inhibition on BCSCs by using a well-established AKT inhibitor, AZD5363 (Capivasertib). Figure 2A shows our confocal observation, which indicated that Mitofusin 1 could be dramatically down-regulated by AZD5363, but not FIS 1. This observation was consistent with the raw data of mean markers expression obtained from flow cytometry (Fig. 2B). Those results indicated that the balance of mitochondrial fusion and fission in BCSCs could be disturbed by AZD5363. To facilitate our investigation, we then performed MitoTracker orange staining to indicate of mitochondrial activity, as well as Hoechst 33342 double staining on BCSCs treated with different concentrations of AZD5363. Figure 2 C, D, and E show that the intensity of MitoTracker orange staining can be dramatically decreased by AZD5363.

The absence of mitochondrial function is considered a hallmark of apoptosis, and recent studies showed that proteins involved in mitochondrial fission and fusion also actively participate in apoptosis induction. Next, we determined the apoptosis induction activity of AZD5363 on BCSCs. We developed a MitoTracker orange/annexin V-FITC co-staining assay, and cells with lower staining of MitoTracker orange and higher expression of annexin V were gated out as described apoptotic cells as showed in Fig. 2F and G. The percentage of apoptotic death cells was 1.21%±0.06, 1.6%±0.43, 7.3%±2.05, and 12.8%±1.96, when treated with 0, 1, 2, and 5μM of AZD5363, respectively. To validate our finding, we also performed an annexin V-FITC/Hoechst 33342 double-staining assay. As showed in Fig. 2H, fluorescence intensity of both annexin V and Hoechst 33342 were increased by AZD5363.

### Stemness increased in stem cell spheres by doxorubicin could be inhibited by AZD5363, and AZD5363 re-sensitized stem cell spheres to doxorubicin

**Fig. 3:**
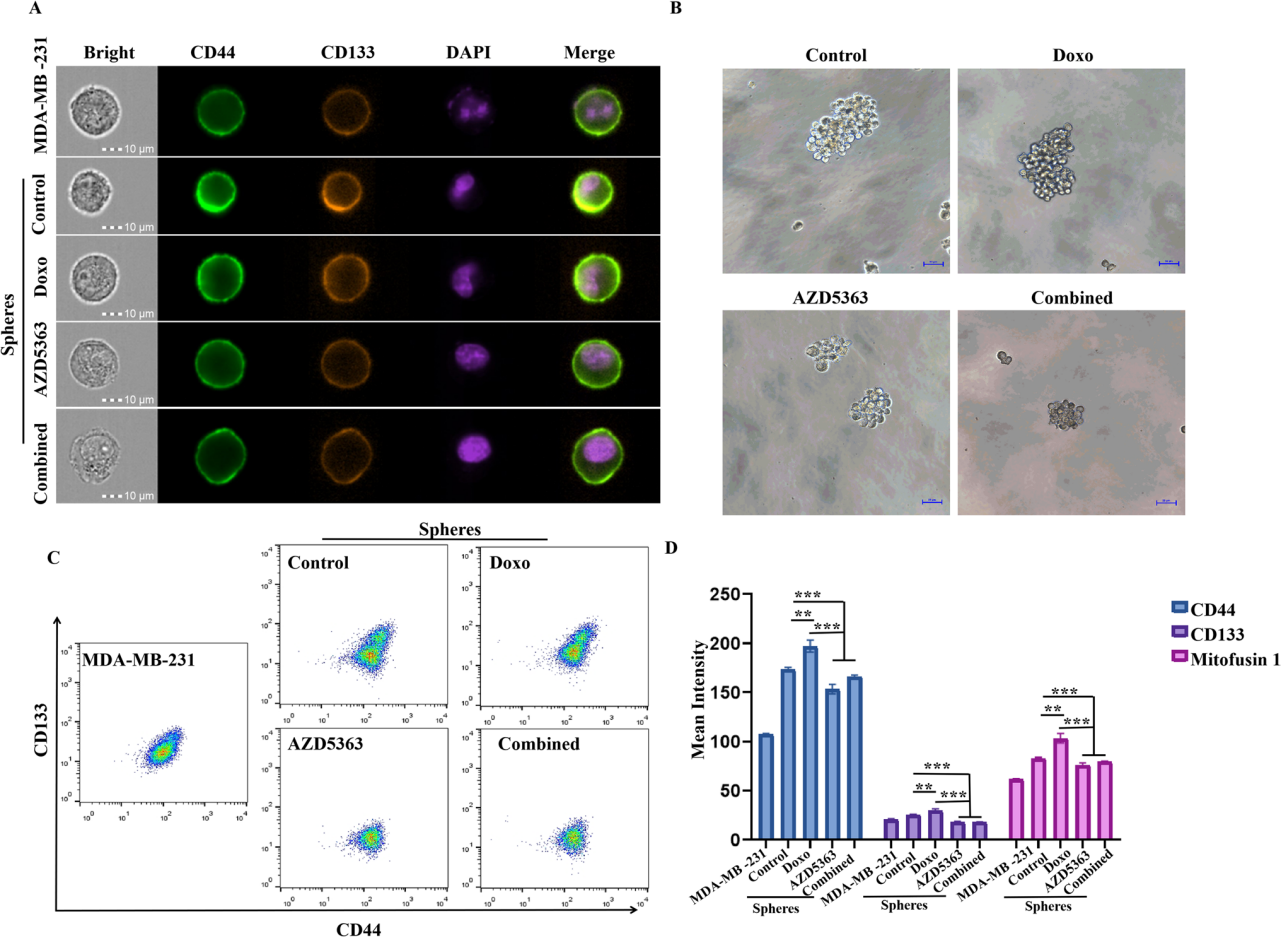
Suppression of stemness in stem cell spheres by AZD5363. **A** Expression of stem cell markers, CD133 and CD44 was detected by imaging flow cytometry. **B** Images of stem cell spheres treated with doxorubicin (Doxo, 50nM), AZD5363(5 μM), and Doxo plus AZD5363. **C**, **D** Dot plots and calculated mean intensity from flow cytometry detection displayed that CD133, CD44 and mitofusin expression could be increased by doxorubicin, and inhibited by AZD5363. * *p* < 0.05, ** *p* < 0.01, *** *p* < 0.001, *n*=3

Doxorubicin has long been used as anti-cancer agents in the treatment of triple-negative breast cancer. Here, we used doxorubicin to evaluate the chemo-sensitivity of BCSCs. The concentrations of doxorubicin (Doxo) ranged from 0 to 600nM (Fig. 1J), and we chose 50 nM (the inhibition rate towards stem cell spheres was 5%) as the experimental concentration both in the presence and absence of 5 μM AZD5363. Recent studies suggested that doxorubicin could promote stemness in carcinoma cells (Karabicici et al., 2021). Accordingly, we first examined stemness in BCSCs by detecting CD133 and CD44, two well-established stemness markers, treated before and after Doxo. Figure 3A shows that, in combination with AZD5363, the CD133 and CD44 expression induced by Doxo (50 nM) in BCSCs was decreased. Figure 3B showed the morphology of BCSCs that cultured in low-attachment 6-well plates when treated with different groups. As we displayed, the combination of AZD5363 with Doxo could significant inhibit spheres formation. Moreover, dot plots and the mean intensity from flow cytometry indicated that stemness could be enhanced by Doxo, and decreased when in combination with AZD5363 (Fig. 3C and D).

**Fig. 4:**
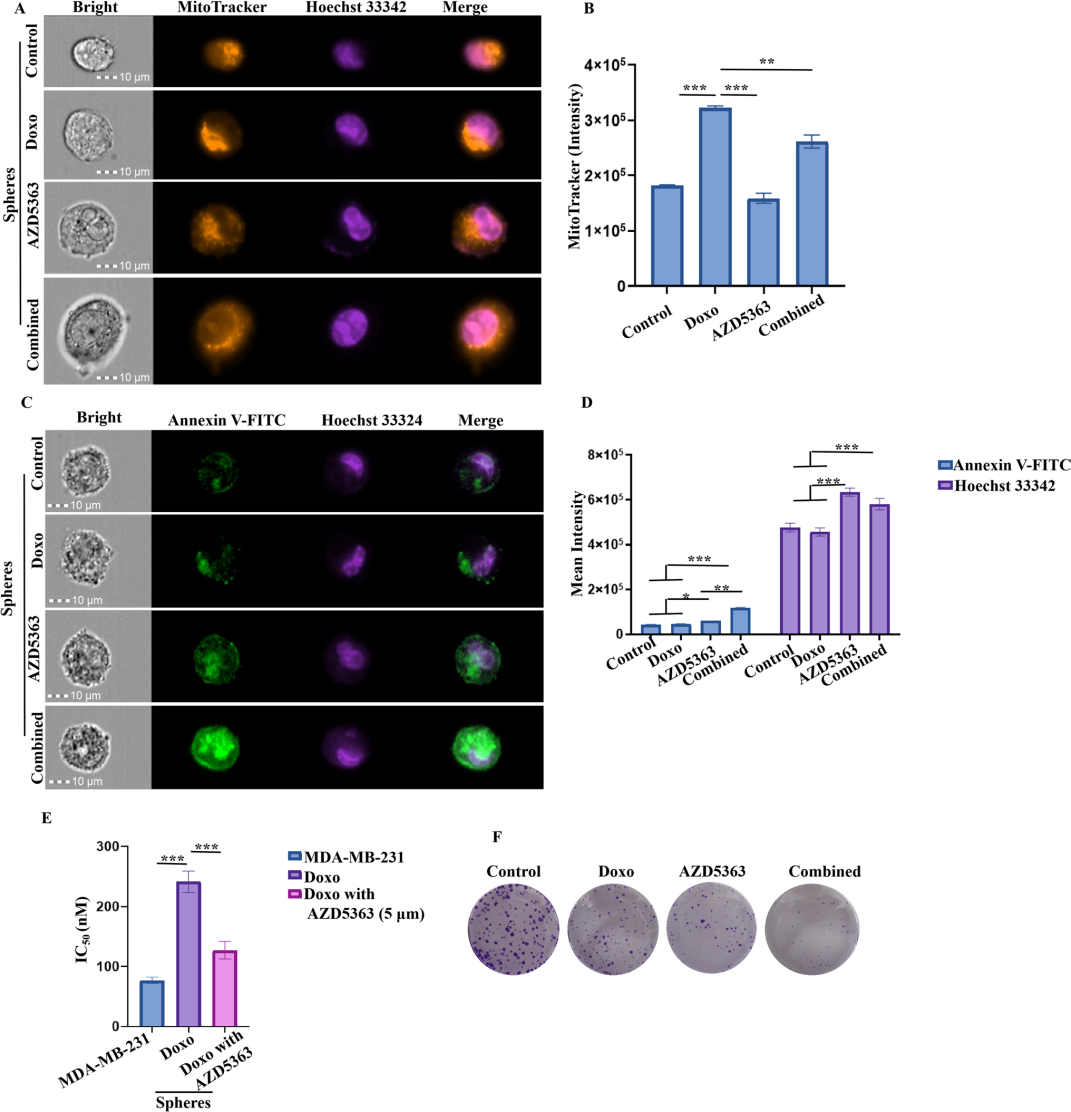
Sensitizing breast cancer stem cells to doxorubicin by AZD5363. **A**, **B** Imaging flow cytometry displayed that MitoTrancker orange staining was enhanced in doxorubicin treated stem cells and could be inhibited by AZD5363. **C**, **D** Annexin V-FITC and Hoechst 33342 double staining assay was used to measure apoptotic death in stem cells. The annexin V positive apoptotic cells was increased in doxo and AZD5363 combination group. **E** IC50 value was measured by CCK8 assay, the results indicated that AZD5363 could up-regulate sensitivity of stem cells to doxorubicin. **F** Colony formation detection of stem cells treated with doxorubicin (Doxo, 50nM), AZD5363 (5 μM), and Doxo plus AZD5363. * *p* < 0.05, ** *p* < 0.01, *** *p* < 0.001, *n*=3

We then focused on the impact of Doxo in combination with AZD5363 on mitochondrial activity and apoptosis in BCSCs. Our results in Fig. 4 showed that, MitoTracker orange intensity was markedly increased after Doxo was administered in BCSCs, and Doxo-increased mitochondrial activity could be suppressed by AZD5363(Fig. 1A, and B). Furthermore, in combination with ZAD5363, Doxo-induced apoptotic cell death was significantly enhanced (Fig. 4C and D). Moreover, the IC_50_ value of Doxo towards MDA-MB-231 cells and BCSCs was also detected by CCK8 assay. As showed in Fig. 4E, IC_50_ value was 76.7±5.1 nM in MDA-MB-231 cells, 240.8±18.1 nM in BCSCs, and 126.8±14.6 nM in BCSCs in the presence of 5 μM AZD5363. A colony formation assay confirmed that AZD5363 could promote the chemo-sensitivity of BCSCs to Doxo as we showed in Fig. 4F.

Collectively, these findings strongly supported that AZD5363 could target mitochondrial dynamics by regulation of Mitofusin in BCSCs, and enhance the chemo-sensitivity of 3D cultured stem cell spheres to doxorubicin.

## Discussion

To date, chemotherapy remains one of the most important treatments against triple-negative breast cancer. However, breast cancer stem cells (BCSCs) have been suggested to contribute to drug resistance and cancer recurrence, which are severe obstacles for chemotherapy to treat breast cancer. Targeting these processes might lead to novel anti-cancer therapies. Accumulating evidence has indicated that mitochondrial dynamics may represent attractive therapeutic avenues to target CSCs for a variety of tumor types (Pei et al., 2018; Civenni et al., 2019; Cieśla et al., 2021; Sessions and Kashatus, 2021; Weiner-Gorzel and Murphy, 2021). In this study, we demonstrated that mitochondrial dynamics were enhanced in 3D cultured triple-negative MDA-MB-231 breast cancer stem cell spheres (BCSCs). In addition, we showed that an AKT inhibitor could regulate Mitofusin expression, and interfere, with mitochondria function, thereby decreasing stemness and increasing chemo-sensitivity in BCSCs.

Mitochondrial dynamics are regulated by two opposed processes, namely mitochondrial fusion and fission. Several proteins, such as Drp1, FIS1, Bax, and Mitofusins, change in activity and might link the mitochondrial fission/fusion events with processes such as alterations in the mitochondrial membrane potential, apoptosis, necrosis, cell cycle arrest, and malignant growth (Horbay and Bilyy, 2016). Therefore, interfering with mitochondria biology and function may be regarded as a useful approach to targeting BCSCs. In this study, we have evaluated the mitochondrial fission protein FIS 1, and mitochondrial fusion protein Mitofusin 1expression in BCSCs by using flow cytometry and confocal microscopy. According to our observations, FIS 1 and Mitofusin 1 were all markedly upregulated in BCSCs compared with 2D cultured MDA-MB-231 cells. Additionally, mitochondrial function, which we observed by Mitotracker orange staining, was also increased in BCSCs. These results indicate that mitochondrial dynamics and functions could be enhanced in breast cancer stem cells.

The AKT pathway plays a central role in tumorigenesis, and its abnormal activation contributes to self-renewal and differentiation in BCSCs. (Bozorgi et al., 2015). In previous studies, AZD5363, a known AKT inhibitor was reported to suppress anti-cancer-agent-induced stemness in breast cancer cell lines. In our study, we proved that AZD5363 could significantly inhibit Mitofusin1 in BCSCs (but not FIS 1), indicating that mitochondrial fusion in BCSCs might be suppressed by AZD5363. A similar trend was also observed for the expression of CD44 and CD133 in BCSCs (two well-established markers of stemness) after treatment with AZD5363. Some studies reported a possible direct correlation between increased mitochondrial fusion and the chemo-resistance of tumor cells (Wang et al., 2022). In our study, the IC_50_ value of doxorubicin, a common chemo drug used against TNBC, was significantly lower when combined with AZD5363 on BCSCs. Moreover, apoptotic death in BCSCs induced by Doxo was also dramatically increased by adding AZD5363. These results indicate that AKT inhibition could suppress stemness, and re-sensitize BCSCs to chemo drugs by disturbing the balance of mitochondrial dynamics.

## Conclusion

Taken together, our study suggested that mitochondrial dynamics and functions could be enhanced in 3D cultured breast cancer stem cell spheres, and that the AKT inhibitor AZD5363 has the potential to suppress stemness, and re-sensitize BCSCs to chemo drugs by regulating mitochondrial fusion. These findings also support the future pursuit of AZD5363 as a therapeutic agent for BCSCs.
